# Activation of the ROCK/MYLK Pathway Affects Complex Molecular and Morphological Changes of the Trabecular Meshwork Associated With Ocular Hypertension

**DOI:** 10.1167/iovs.65.10.17

**Published:** 2024-08-08

**Authors:** Chia-Chen Hsu, Fang-Pai Lin, Hao-Chen Tseng, Pin Kuan Ho, Yi-Hsun Chen, Yann-Guang Chen, Da-Wen Lu, Yi-Hao Chen, Jian-Liang Chou, Hsin-Chih Chen, Yu Chuan Huang

**Affiliations:** 1 Department of Ophthalmology, Tri-Service General Hospital, National Defense Medical Center, Taipei, Taiwan; 2 School of Pharmacy, National Defense Medical Center, Taipei, Taiwan; 3 School of Dentistry, National Defense Medical Center, Taipei, Taiwan; 4 Biomedical Technology and Device Research Laboratories, Industrial Technology Research Institute, Hsinchu, Taiwan; 5 Department of Research and Development, National Defense Medical Center, Taipei, Taiwan

**Keywords:** primary open-angle glaucoma (POAG), intraocular pressure (IOP), microbead-induced, ocular hypertension (OHT), Rho-kinase inhibitor (ROCK), trabecular meshwork (TM), aqueous humor (AH) outflow, myosin light chain, myosin light chain phosphatase (MLCP), myosin light chain kinase (MYLK), early/late-onset POAG, whole transcriptome sequencing, ITRI-E-(S)-4046

## Abstract

**Purpose:**

The Rho-associated protein kinase and myosin light chain kinase (ROCK/MYLK) pathway undeniably plays a pivotal role in the pathophysiology of primary open-angle glaucoma (POAG). In our study, we utilized both ocular hypertension (OHT) rabbit models and clinical investigations to gain invaluable insights that propel the development of novel treatments targeting proteins and genes associated with the trabecular meshwork (TM), thereby offering promising avenues for the management of POAG.

**Methods:**

Following microbead injections into the anterior chamber of the ocular cavity of rabbits, we observed elevated histiocyte numbers and immune scores for MYLK-4/ pMLC-2, alongside a reduction in the void space within the TM. Notably, treatment was performed with 0.1% ITRI-E-(S)-4046, a compound with dual kinase inhibitor (highly specific inhibitor of ROCK1/2 and MYLK4), significantly reduced intraocular pressure (IOP; *P* < 0.05) and expanded the void space within the TM (*P* < 0.0001) compared with OHT rabbits. In clinical investigations, we utilized whole transcriptome sequencing to analyze gene expression specifically related to the TM, obtained from patients (5 early-onset and 5 late-onset) undergoing trabeculectomy.

**Results:**

Our findings revealed 103 differential expression genes (DEGs) out of 265 molecules associated with the Rho family GTPase pathway, exhibiting a *P* value of 1.25E-10 and a z-score of −2.524. These results underscore significant differences between the early-onset and late-onset POAG and highlight the involvement of the ROCK/MYLK pathway.

**Conclusions:**

These findings underscore the critical involvement of the ROCK/MYLK pathway in both OHT-related and different onsets of POAG, providing valuable insights into the TM-related molecular mechanisms underlying the disease.

Glaucoma remains the second leading cause of blindness worldwide, with more than 100 million patients expected to be affected by 2040.^1^ Primary open-angle glaucoma (POAG), the most prevalent form of glaucoma, often manifests with elevated intraocular pressure (IOP) and is characterized by distinct optic disc damage and visual field defects, leading to optic nerve damage and potential vision loss. Early-onset POAG, although less common, presents at a younger age (3 to 40 years) and may be associated with genetic factors. The trabecular meshwork (TM) plays a crucial role in regulating aqueous humor (AH) outflow resistance and maintaining normal IOP. The TM-related outflow pathway accounts for approximately 70% to 95% of AH drainage (up to 90% in older adults).^2^^,^^3^ Dysfunction in the TM leads to decreased AH drainage facility and elevated IOP, which is considered the only modifiable risk factor in treating POAG.^4^

One of the key signaling pathways implicated in POAG is the Rho family GTPase pathway, which has an impact on regulating cell shape, motility, and contraction.^5^ One study investigated the effect of IOP on AH outflow resistance through in vitro mechanical stimulation. By establishing an IOP of 50 mm Hg and monitoring the activation of Rho GTPase, an increase in AH efflux resistance was observed.^6^ As depicted in Figure 1, particularly through the activation of the Rho-associated protein kinase and myosin light chain kinase (ROCK/MYLK) pathway induces smooth muscle contraction by phosphorylated myosin light chain (pMLC) within the TM, subsequently leading to elevated IOP, a hallmark of POAG. In addition, myosin light chain phosphatase (MLCP) acts to dephosphorylate the myosin light chain (MLC) and reverse the effects of MYLK, promoting relaxation of the TM and facilitating AH outflow, which helps to regulate IOP.^7^^,^^8^ The balance between phosphorylated (active) and non-phosphorylated (inactive) MLC^9^^,^^10^ in the TM of the eye is dynamically achieved by two enzymes: (i) MYLK, a Ca^2+^-dependent enzyme, phosphorylates MLC, thereby activating the contractile machinery in smooth muscle cells; and (ii) MLCP, which typically acts to reverse the effects of ROCK by dephosphorylating the myosin light chain (pMLC).

**Figure 1.**
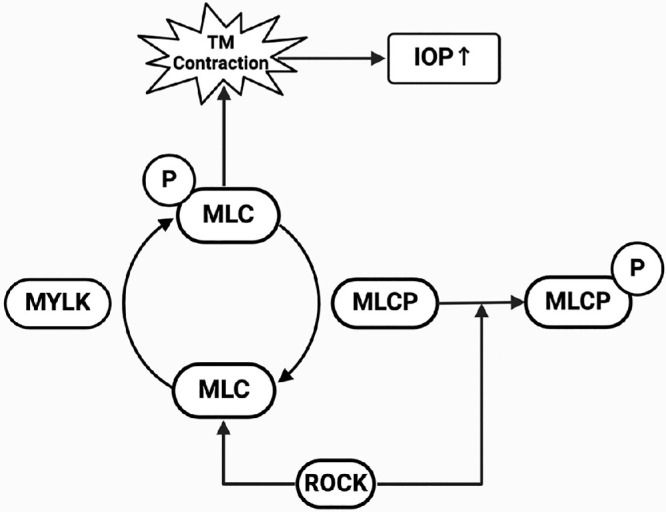
Introducing a simplified plot structure for our study on microbead-induced OHT in rabbits, focusing on the activation of ROCK/MYLK pathway-associated proteins, leads to the phosphorylation of myosin light chain (p-MLC), increasing contraction in the TM and subsequently raising IOP. Conversely, myosin light chain phosphatase (MLCP) reverses this effect by dephosphorylating MLC, thereby reducing TM contraction and helping to lower IOP. The dynamic expression of ROCK/MYLK pathway-associated proteins are critical markers in the molecular mechanisms of OHT and the development of anti-glaucoma drugs.

In recent years, the Rho family GTPase has emerged as a new target for regulating AH outflow resistance.^11^^–^^13^ In the last decade, ROCK inhibitors that target the TM are effective in patients with POAG, ripasudil (K115) in Japan and netarsudil (AR-13324) in the United States, have been marketed for glaucoma treatment.^14^^,^^15^ Additionally, we have developed a series of dual kinase inhibitors (ITRI-E-212, ITRI-E-(S)4046, etc.). Among them, ITRI-E-(S)4046 (MW = 379.3), which is a compound characterized by its dual kinase inhibition capabilities, stands out as a novel and highly specific inhibitor of both ROCK1/2 and MYLK4 (100% for ROCK1/2 and 99.5% for MYLK), that reduces IOP in ocular hypertension (OHT) animals with low incidence of ocular hyperemia.^16^^,^^17^ ROCK inhibition is a pharmacological mechanism attracting medical development in glaucoma.^18^

Our study aims to elucidate the involvement of the ROCK/MYLK pathway by examining protein variations in OHT model of New Zealand White (NZW) rabbits and the gene expression pattern in clinical patients on different onsets of POAG. This approach helps us to understand the gene expression and morphological changes in the TM and its role in the pathogenesis of glaucoma,^19^^,^^20^ and also provide a platform to evaluate the efficacy of different antiglaucoma drugs.^21^^,^^22^ Understanding the involvement of the ROCK/MYLK pathway with other gene expression patterns in patients with different onset POAG can provide valuable insights into the molecular mechanisms underlying the disease.

## Materials and Methods

### OHT Model of NZW Rabbits

All animal procedures conducted in this study followed the UK Animals (Scientific Procedures) Act 1986 (protocol No. 5), and guidelines in the Association for Research in Vision and Ophthalmology Statement for the Use of Animals (see Supplementary Methods S1A, Rabbits’ handling). Additionally, the study protocol was approved by the Animal Care and Use Committee of the National Defense Medical Center (approval number IACUC-21-222). Exclusively male rabbits were utilized throughout the experiments. IOP was obtained in each eye using a Model 30 Pneumatonometer (Reichert, NY, USA), an applanation tonometer (Supplementary Fig. S1, Supplementary Methods S1B). To induce OHT, a solution containing 100 µL of irregular-shaped superparamagnetic iron oxide microbeads (10 µm, BioMag; Polysciences, Inc., Warrington, PA, USA) suspended in deionized water and stored at 4°C (50 mg/mL) was utilized. The left eye (OS) served as a contralateral control eye (see Supplementary Methods S2, The procedure for inducing OHT in rabbits). A customized toroidal (TATONG, New Taipei City, Taiwan), donut-shaped neodymium magnet, with external and internal diameters of 20 mm and 16 mm (1.43–1.48 tesla), respectively, was placed in the center of the eye to fit around the rabbit’s eyeball. IOP measurements were performed before injection and on days 1, 2, 3, 4, 7, 10, and 14, at approximately the same time (between 16:00 and 17:00), to minimize diurnal variation in IOP.

### Chemicals and Test Compounds

ITRI-E-(S)4046 2HCl (molecular weight = 379.29 and free base = 306.3) is a highly hydrophilic amino-pyrazole derivative that acts as a potent inhibitor of ROCK (half-maximal inhibitory concentration [IC50] = 3.2 ± 0.5 nM). Additionally, it exhibits selective inhibition of MYLK with an IC50 of 25.6 nM by using the KINOME scan screening platform.^17^ The ophthalmic solution also contained a surfactant (Nonoxynol-9) to enhance the ocular penetration of ITRI-E-(S)4046. AR-13324 is a clinically approved treatment of POAG and OHT that we are currently using as comparison. Rhopressa (Aerie Pharmaceuticals Inc., Irvine, CA, USA), a ROCK inhibitor currently used for the treatment of POAG and OHT, was used for comparison. AR-13324 has an IC50 of 10 nM for Rho-kinase.^12^

### IOP-Lowering Effect of Test Compounds in the OHT Model of NZW Rabbits

The OHT model of NZW rabbits was used to examine the IOP-lowering effect of 0.02% AR-13324, 0.05% ITRI-E-(S)4046, and 0.1% ITRI-E-(S)4046, compared to the vehicle alone (0.05% boric acid, 4.7% mannitol, and 0.125% nonoxynol-9 in double-distilled water). On day 3, after magnetic microbead injection, when IOP was significantly elevated, the OD was treated with one of the ROCK inhibitors or the control vehicle. Each treatment as a single administration of 35 microliters of 0.02% AR-13324, 0.05% ITRI-E-(S)4046, 0.1% ITRI-E-(S)4046, or the control vehicle into the OD once daily for 2 consecutive days (*n* = 7 in each group). In our study, we utilized 2D tissue slides to define pore size, evaluating the void space following treatment with 0.1% ITRI-E-(S)4046. This assessment is essential for analyzing TM morphology in two primary groups: the control group and the microbead-induced OHT group.

### Expression of p-MLC-2 and MLCK4 in the OHT Model of NZW Rabbits

On day 14, post-magnetic microbead-injected, TM tissue was dissected from the anterior segment and fixed. Rabbit polyclonal antibodies specific for pMLC-2 (Thr18/Ser19, 1:200; Cell Signaling Technology, Taipei, Taiwan) and MYLK-4 (1:200, Antibody [NBP1-80761]; Novus Biologicals, Centennial, CO, USA). We utilized digital imaging software for automatic segmentation and quantification of stained regions, effectively differentiating them from the background based on color thresholds to tag pMLC-2 and MYLK-4 in brown, respectively. The calculated method is mainly based on morphological features and consistent threshold (see Supplementary Fig. S2). An alternative approach to the International Harmonization of Nomenclature and Diagnostic Criteria (INHAND) is detailed in Supplementary Methods S3 and has been applied to Supplementary Figures S4 and S5. The protocol for hematoxylin and eosin (H&E) examination was executed on the OHT model of NZW rabbits (see Supplementary Methods S4).

### Patients

A total of 10 patients with POAG (for patients’ information, see Table 1) were enrolled to explore differential expression genes (DEGs) by RNAseq. The patients were recruited during their routine trabeculectomy procedure at the tri-service general hospital (Taipei, Taiwan). In this study, we conducted a comparison between two subgroups: (1) five individuals (age > 60 years, Visual Field Index [VFI] > 87%) diagnosed with late-onset POAG, as the “reference” group in our study; (2) five individuals (aged between 20 and 40 years) diagnosed with non-family-related early-onset POAG. Patients with acute, uveitic, and congenital glaucoma were excluded. This study was approved by the Institutional Review Board of the Research Ethics Committee of Tri-Service General Hospital (Protocol A202205051), and all patients provided written consent.

**Table 1.** The Demographic and Clinical Characteristics of the Study Participants, Providing an Overview of the Age Distribution and Disease Severity Between the Control and Case Groups

Patient ID	Gender	Age	RIN	RNFL	VFI	C/D Ratio
(A) Cases (*n* = 5) <40 years old
B68	M	20	7.22	73	91	0.35
C33	F	35	8.3	63	65	0.79
C50	F	31	8.09	86	99	0.75
C67	F	27	8.04	88	94	0.59
D22	F	35	8.58	72	95	0.75
Average	20% M	29.6 ± 6.3	8.05 ± 0.5	76.4 ± 10.5	88.8 ± 13.6	0.65 ± 0.2
(B) References (*n* = 5) >60 years old						
F37	F	75	7.4	70	80	0.41
G40	F	75	7.43	101	74	0.57
G87	M	82	7.73	61	92	0.42
H12	F	75	8.46	105	100	0.08
H27	M	75	7.48	63	78	0.65
Average	40% M	76.4 ± 3.1	7.7 ± 0.4	80 ± 21.3	84.8 ± 10.8	0.43 ± 0.22

(A) Case = patients with early-onset (hereditary, age < 40 y) POAG (*n* = 5); (B) control = patients with late-onset (age-related, age > 60 y) POAG (*n* = 5). The cup/disc ratio (CDR) was measured by fundas color; RNFL (retinal nerve fiber layer) was measured by optical coherence tomography; VFI (visual field index) was obtained by standard automated perimetry. This study performed whole transcriptome sequencing (mRNAseq) of TM tissue from patients with POAG undergoing trabeculectomy.

### RNAseq and Data Preprocessing

Total RNA extraction was performed in TRIzol reagent according to the manufacturer’s protocol of TANBEAD (Taiwan Advanced Nanotechnology Co., Ltd., Taiwan). Subsequently, RNA purification, quantification, and integrity were assessed using Qbit (Thermo Scientific) and Tape station 4150 (Agilent Technologies). Once the total RNA amount was >25 ng and RIN >7, libraries were constructed using the TruSeq Stranded mRNA Sample Prep Kit (Illumina) and whole transcriptome sequencing was executed on the Illumina Nextseq550 platform. After, the raw reads obtained by sequencing were filtered. We utilized CLC Genomics Workbench 23.0.5 and Ingenuity Pathway Analysis (IPA) for bioinformatic analysis (see Supplementary Methods S5, The bioinformatic analysis of RNAseq data).

### Statistics

Statistical analyses were performed using GraphPad PRISM software, version 9. The Mann-Whitney *U* test was used for comparing two independent samples, ANOVA was used to analyze differences among group means, followed by Tukey’s test for pairwise comparisons where applicable. The Kruskal-Wallis test assessed the distribution of continuous variables across multiple groups. IOP levels are expressed as the mean ± standard deviation (SD). Statistical significance was determined using a two-tailed test with a significance threshold set at *P* < 0.05.

## Results

### IOP Profile in the Microbead-Induced OHT Model of NZW Rabbits

**Figure 2.**
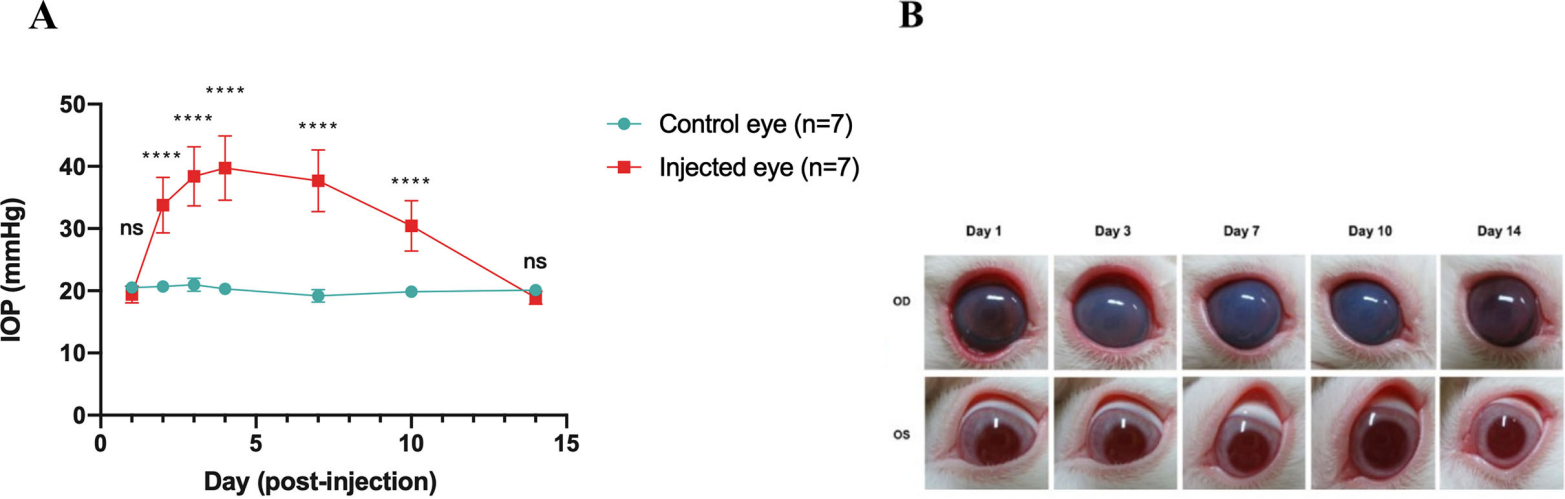
IOP is measured at multiple time points to monitor changes over time. Mean IOP in microbead-injected eyes and non-injected eyes. (**A**) IOP elevated acutely on day 1 following the injection of magnetic microbeads into the eye. The IOP reaches plateau around day 2 post-injection and gradually decreased after day 7 post-injection. The notation **** (*P* < 0.0001) indicates that the differences in IOP between the microbead-injected eyes (*n* = 7) and the control eyes (*n* = 7) are statistically significant as determined by a 2-way ANOVA, suggesting a direct impact of the microbeads on IOP. (**B**) External eye photography observed on days 1, 3, 7, 10, and 14 post-injection of magnetic microbeads in the right eye (OD) and control eye (OS).

The difference in IOP (ΔIOP) between the microbead-injected and control eyes was significant from the first day to the 10th day following injection (*P* < 0.05, *n* = 7; Fig. 2A). During this observational period, microbead injection led to a ∆IOP of 14.6 to 30.8 mm Hg and a mean elevation of maximal ∆ IOP of 20.95 ± 6.17 mm Hg. During the injection of the microbeads, a rod-shaped magnet was placed parallel to the iridocorneal angle, but unfortunately, the microbeads were unevenly distributed and led to their accumulation at the focal area only (Supplementary Fig. S3A). Consequently, we customized a toroidal, donut-shaped, neodymium magnet which was used to attract the microbeads into the iridocorneal angle with an even 360-degree distribution (see Supplementary Fig. S3B). After 14 days, these eyes appeared to be clear, which demonstrated a subsequent reduction in IOP (Fig. 2B).

### IOP-Lowering Effect of Test Compounds in the OHT Model of NZW Rabbits

**Figure 3.**
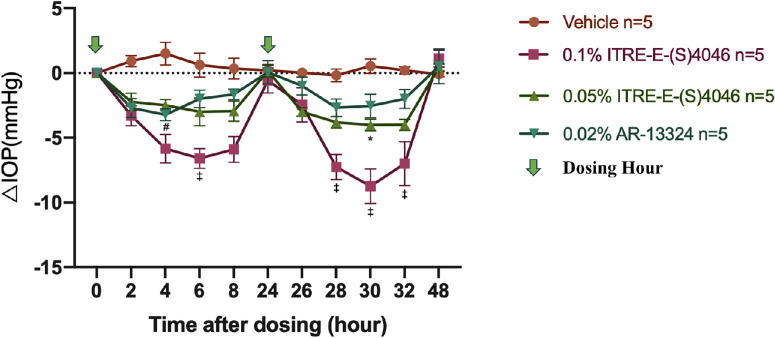
IOP lowering effect of vehicle, and three topical administration distinct treatment groups (included 0.05% ITRI-E-(S)4046, 0.1% ITRI-E-(S)4046, and 0.02% AR-13324, *n* = 5 in each group) were given to each microbead-injected OHT eye once daily for 2 consecutive days (at 0 and 24 hours). The ∆IOP was measured at 0, 2, 4, 6, 8, 24, 26, 28, 30, 32, and 48 hours. The ∆ IOP (mm Hg) was the change of IOPs from time zero IOP, vertical bars represent means ± standard error of the mean (SEM) of data obtained from 5 rabbits by 2-way ANOVA. The 0.1% of ITRI-E-(S)4046 was significantly more effective in reducing IOP compared to 0.05% ITRI-E-(S)4046 (*, *P* < 0.05); 0.1% ITRI-E-(S)4046 also showed a significantly greater IOP reduction compared to 0.02% AR-13324 (‡, *P* < 0.05); 0.02% AR-13324 significantly lowered IOP compared to the vehicle control (#, *P* < 0.05).

The maximum effect of 0.02% AR-13324 was observed at 4 hours after the first dose, resulting in an IOP decrease from 36.38 ± 5.26 mm Hg to 33.16 ± 4.38 mm Hg. The ∆IOP was significantly different between 0.02% AR-13324 and the control vehicle from 2 to 6 hours after the first dose and 4 to 8 hours after the second dose (*P* < 0.05, *n* = 5). IOP gradually returned to baseline after 24 hours of treatment (Fig. 3, Table 2). After the first dose, 0.05% and 0.1% ITRI-E-(S)4046 resulted in a maximum IOP reduction of 4.5 ± 1.5 mm Hg (13.4%) and 6.6 ± 1.7 mm Hg (18.2%), respectively. After the second dose, the reduction was 5.6 ± 0.6 mm Hg (14.5%) and 8.74 ± 3.0 mm Hg (24.0%), respectively (see Table 2). The maximum effect of both 0.05% ITRI-E-(S)4046 and 0.1% ITRI-E-(S)4046 was observed 6 hours after the second dose with the peak effect of each dose at 6 hours post instillation. In contrast, the second doses of 0.05% and 0.1% ITRI-E-(S)4046 showed superior hypotensive efficacy compared with the initial doses. The second dose of 0.1% ITRI-E-(S)4046 reduced the IOP by 8.74 ± 3.0 mm Hg between 4 and 6 hours post topical installation, compared to 6.6 ± 1.7 mm Hg after the first dose (see Fig. 3). Among the 3 agents, 0.1% ITRI-E- (S)4046 was the most effective at reducing IOP in rabbits with OHT (*P* < 0.05, *n* = 5), achieving more than a 2-fold IOP decline compared to 0.02% AR-13324.

**Table 2.** The Percent of △maxIOP of Microbead-Induced Rabbits Were Assessed Onto the Experimental OD After Treating With Rho-Kinase Inhibitors, Netarsudil (AR-13324, 0.02% w/v) and ITRI-E-(S)4046 (0.05% w/v and 0.1% w/v) Once Daily for 3 Consecutive Days (at 0, 24, and 48 hours)

	Magnetic Microbead–Induced OHT Rabbit			
Treatment Compound	Dose	△_max_^*^ (IOP, mm Hg) First Dose/Second Dose	△_max_ (IOP%) First Dose/Second Dose	Time_max_ (h) First Dose/Second Dose
Vehicle		0.2 ± 1.0/−0.18 ± 1.1	0/0	24/4
AR-13324	0.02%	−3.2 ± 1.3/−2.68 ± 1.5	−8.9/−7.4	4/4
ITRI-E-(S)4046	0.05%	−4.5 ± 1.5/−5.6 ± 0.6	−13.4/−14.5	6/6
ITRI-E-(S)4046	0.1%	−6.6 ± 1.7/−8.74 ± 3.0	−18.2/−24.0	6/6

* The ∆max is the maximal hypotensive response calculated from IOP measured at different time points deducted from pretreatment IOP.

The instillation of medication was performed onto the experimental OD, whereas vehicle treatment was performed on the control left eyes (OS). △maxIOP% = (△max IOPOR–△max IOPOS)/(△max IOPOS) x %, to show the IOP lowering effect of test compound in OHT rabbit model.

### Molecular and Morphological Changes of the OHT Rabbit Model With ITRI-E-(S)4046

**Figure 4.**
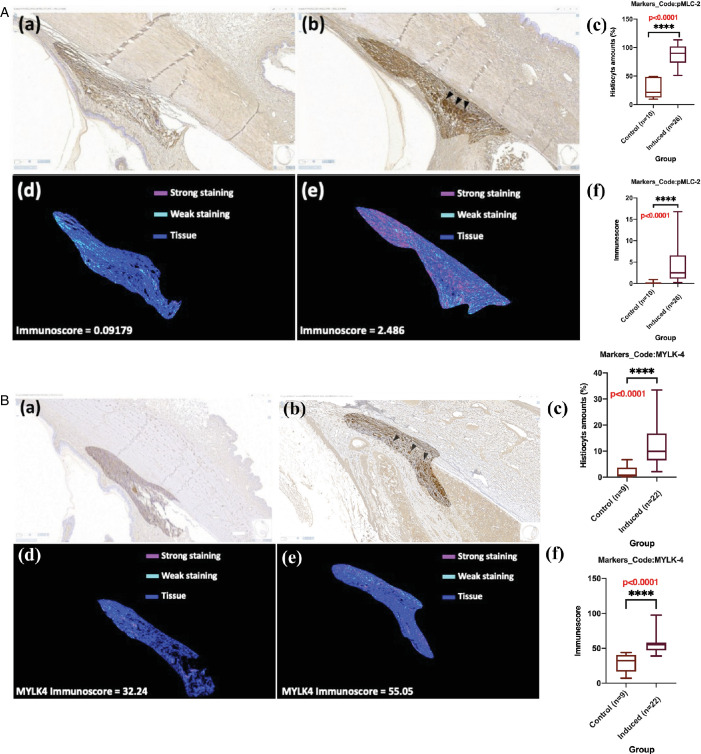
IHC analysis. (**A**) For pMLC-2, in eyes injected with microbeads (*n* = 26), the median of histiocyte count in **a**, **b**, and **c** was found to be approximately 20-fold higher compared to non-induced eyes (*n* = 10), indicating significant differences in histiocytes ratio between the two groups and a severe inflammatory response (****, *P* < 0.0001). Additionally, the median of p-MLC2 immune score in **d**, **e**, and **f** was observed to be approximately 4-fold higher (****, *P* < 0.0001), suggests enhanced enzymatic activity that contributes to increased muscle contraction, as depicted in (**A**). (**B**) For MYLK-4, in eyes injected with microbeads (*n* = 22), the median of histiocyte count in **a**, **b**, and **c** was found to be 12-fold higher (****, *P* < 0.0001) in microbead-injected OHT eyes (*n* = 9), with the median of MYLK-4 immune score in **d**, **e**, and **f** being 1.7-fold higher (****, *P* < 0.0001), as depicted in (**B**). Both parts of this analysis used the Mann-Whitney test, a nonparametric method suitable for comparing two independent samples that may not follow a normal distribution.

**Figure 5.**
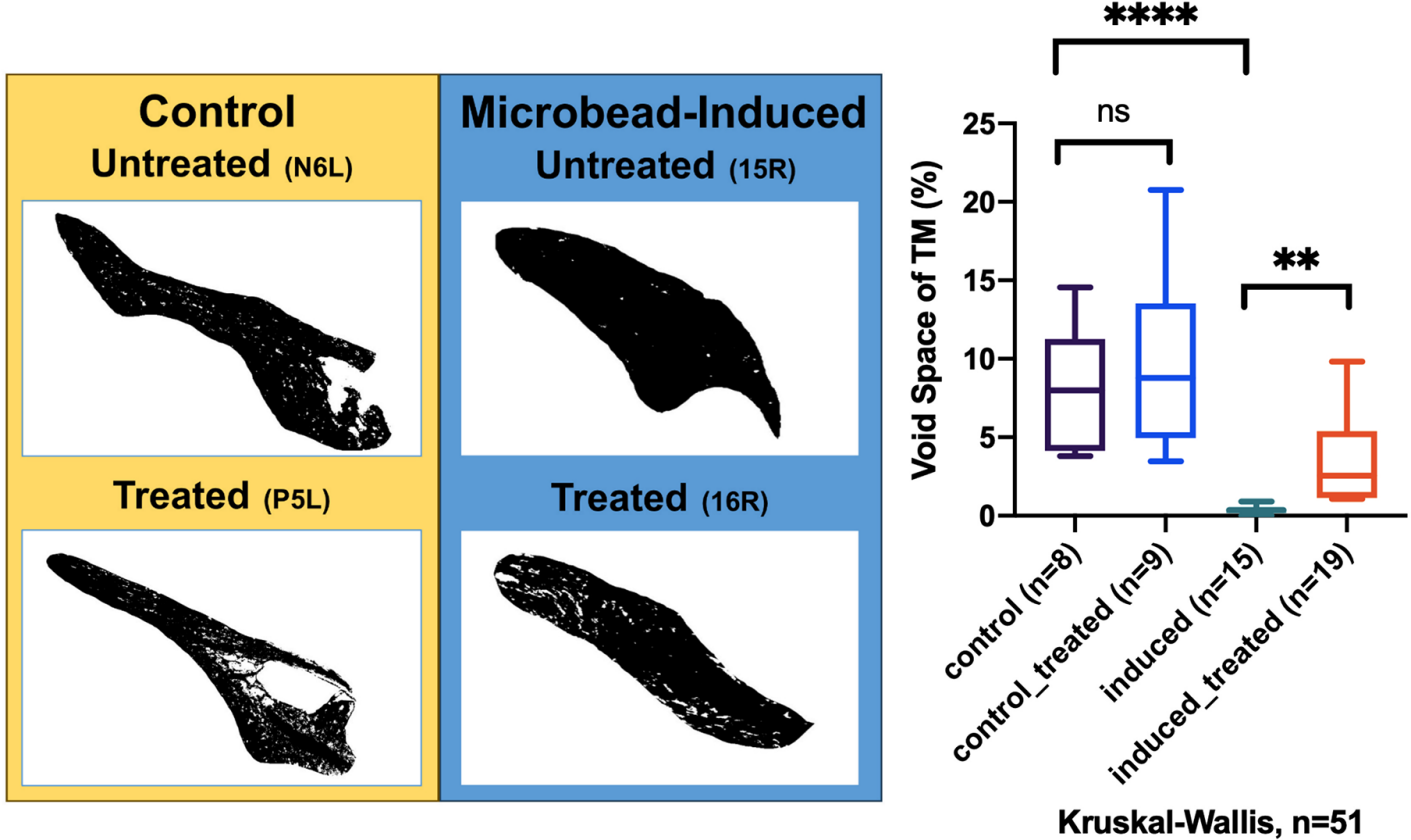
Illustrates the morphological changes, specifically the percentage of the void space, in the TM of the OHT rabbit model. It presents two different experimental groups: the control group (on the left side in color *yellow*, *n* = 8) and the microbead-induced OHT group (on the right side in color *blue*, *n* = 15). On the *yellow box*, representing the control group, no significant differences were observed in the mean rank percentage of void space within the TM compared to administration of 0.1% ITRI-E-(S)4046 (P5L) (*n* = 9). However, prior to administration of 0.1% ITRI-E-(S) 4046 (*upper* in the *blue box*), microbead-induced OHT (*n* = 15) resulted in a significant increase in mean rank of the percentage of void space within the TM compared to the control group (*P* < 0.0001, ****) (*bottom* in the *blue box*). Right after application of 0.1% ITRI-E-(S) 4046 (*n* = 19), the mean rank of the percentage of void space within the TM increased by 3-fold in the microbead-induced OHT group (*bottom one*) (*P* = 0.0011, **) using the Kruskal-Wallis test.

In magnetic microbead-induced OHT rabbits’ eyes, the expression of pMLC-2 and MYLK-4 was significantly elevated compared to contralateral control eyes (see Supplementary Figs. S4, S5). The mean scores for pMLC-2 and MYLK-4 expression were 3.4 and 3.3, respectively, in microbead-injected eyes, whereas they were 0 in the control eyes. Another analysis, shown as Supplementary Method S3 and Supplementary Figure S2, showed that the number of histiocytes and the immune scores for pMLC-2 and MYLK-4 were significantly higher in microbead-injected eyes compared to control eyes (Figs. 4A, 4B). The median number of histiocytes in eyes injected with microbeads was found to be approximately 12 to 20 times higher than in non-induced eyes (*P* < 0.0001; Figs. 4Ac, 4Bc). Additionally, the immune score of pMLC-2 was 4 times higher in microbead-injected eyes compared to noninduced eyes (*P* < 0.0001; see Fig. 4Af); and the immune score for MYLK-4 was 1.7-fold higher than in noninduced eyes (*P* < 0.001; see Fig. 4Bf). We summarized the results of pMLC-2 and MYLK-4 higher expression in TM in resulting TM contraction (check with Fig. 1). In addition, the H&E examination revealed that the magnetic microbeads were mainly localized within the TM (Supplementary Fig. S6A, brown). When no magnetic microbeads were injected, no microbeads were displayed in a similar location (see Supplementary Fig. S6B). Regarding the void space (Fig. 5), the induced eyes (15R) exhibited a significantly lower percentage (1.53 ± 1.06) compared to noninduced eyes (control, N6L, 9.05 ± 1.81, ****, *P* < 0.0001), indicating a substantial reduction due to microbead induction (see Fig. 5). Moreover, the percentage of the void space in treated eyes (16R, 5.85 ± 2.77) was approximately 3.8 times higher compared to microbead-induced eyes (15R), suggesting that 0.1% ITRI-E-(S)4046 demonstrated efficacy as a potential treatment for reversing induced conditions (15R) at 3 days after instillation (**P < 0.001) by expanding the void space. Additionally, administration of 0.1% ITRI-E-(S)4046 (9.75 ± 1.67) in noninduced NZW rabbit eyes (P5L) showed no statistical significance compared with the control (N6L). On day 3, after treatment with 0.1% ITRI-E-(S)4046 in microbead-induced NZW rabbit eyes, the porosity of the TM significantly increased in microbead-induced eyes, with no observed effect in non-induced (control) eyes.

### Pathways Between Early- and Late-Onset POAG in our Clinical Investigation

#### Principal Component Analysis

**Figure 6.**
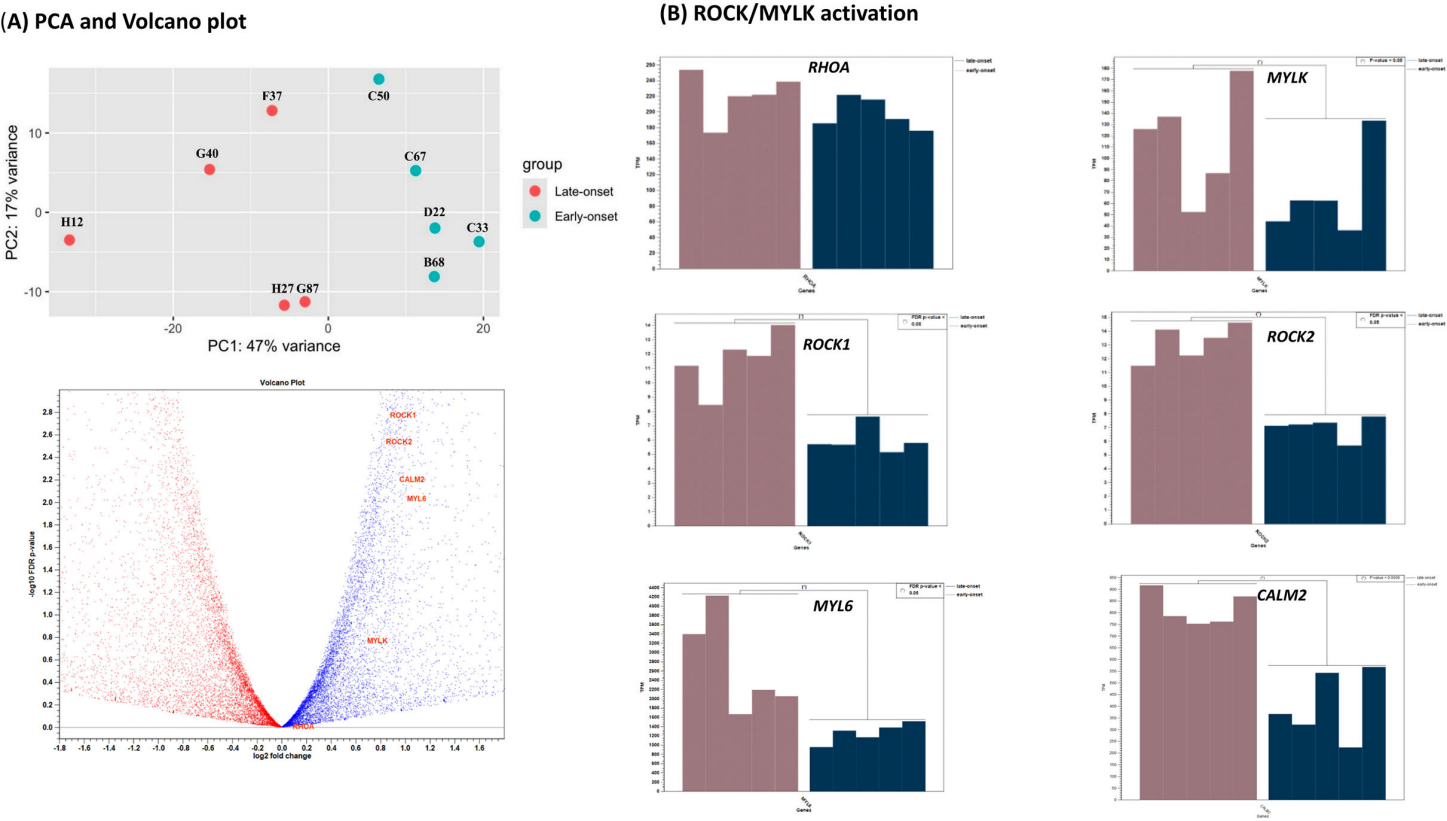
(**A**) In the *upper panel*, the Principal Component Analysis (PCA) plot, based on expression data, reveals differing gene expression dispersion between these two subtypes. The patients with late-onset (*red*) POAG demonstrates higher dispersion in gene expression, whereas the patients with early-onset (green) POAG displays lower dispersion. The Volcano Plot illustrated in the *lower panel* of (**A**), along the y-axis, it plots the negative log of the *P* value against the log fold change between the two groups, helping identify genes that are significantly upregulated or downregulated, whereas genes higher on the y-axis have lower *P* values, indicating higher statistical significance. Genes located further to the left or right on the x-axis represent greater fold changes in expression, the genes involved in the ROCK/MYLK pathways, such as RhoA, ROCK1/2, MYLK, MYL6, and CALM2 are marked in *red* and are all upregulated in patients with early-onset POAG. (**B**) Potential differential expression genes (DEGs) in TM, such as MYL6, MYLK, CALM2, and ROCK1/2 exhibit higher expression in patients with early-onset POAG compared with patients with late-onset POAG. Along the y-axis, transcripts per million (TPM) is utilized to represent the expression levels of various genes.

The case group, with an average age of 29 years, retinal nerve fiber layer (RNFL) thickness of 82, and VFI of 76.6%. The control group, with an average age of 71 years, RNFL thickness of 85, and VFI of 94% (see Table 1). PCA analysis (Fig. 6^7^A, upper panel) revealed differing expression dispersion between the groups, indicating distinct patterns or differences in the expression levels of genes or variables. Late-onset POAG showed greater dispersion in the PCA, suggesting a wider range of molecular factors contributing to this subtype. Conversely, early-onset POAG exhibited lower dispersion, indicating that fewer factors, such as genetic factors, may play a more dominant role in this subgroup.

**Figure 7.**
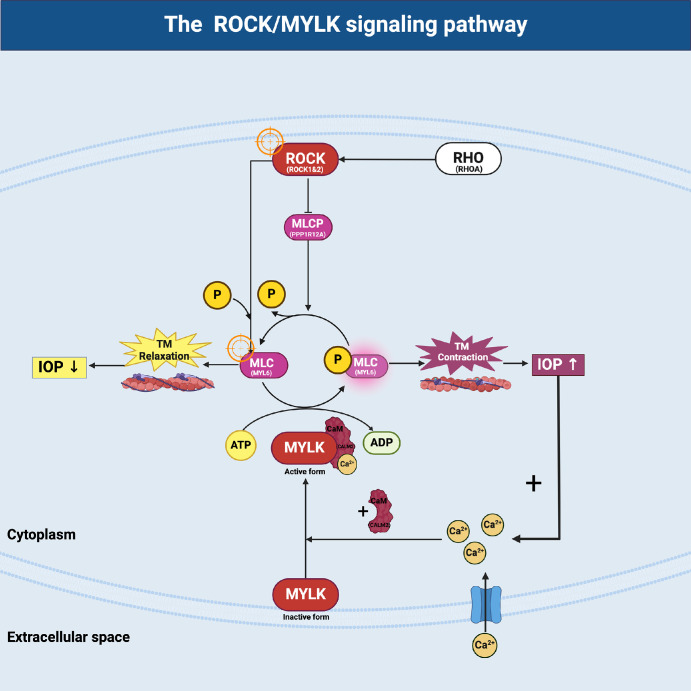
The significantly increased expression of ROCK1 (*P* < 1.16 × 10-4, *n* = 5), ROCK2 (*P* < 2.3 × 10^−4^, *n* = 5), MYLK (*P* < 0.05, *n* = 5), as well as CALM2 (*P* < 0.0006, *n* = 5) in patients with early-onset POAG compared with patients with late-onset POAG is a compelling finding. These results strengthen the hypothesis that alterations in the ROCK/MYLK pathway play a crucial role in the pathology of early-onset POAG through the enhanced activation of phosphorylated myosin light chain (p-MLC). Therefore, the ROCK/MYLK path is activated. In summary, the pathogenic mechanism in patients with early-onset POAG involves a higher activation level of the ROCK/MYLK signaling pathway compared to patients with late-onset POAG, similar to the pathway activation observed in microbead-induced OHT rabbits.

#### TM-Related Genes Between Early- and Late-Onset POAG in our Clinical Investigation

In our supplementary material, Supplementary Figure S7 contains results from the IPA, which is a bioinformatics analysis software. The analysis is based on DEG sets in different onsets of POAG. It helps in identifying key molecules and pathways that are significantly altered in early-onset POAG, especially GTPases, a group of genes/proteins that play a crucial role in these analyzed pathways. The bar chart shows that 103 out of 265 molecules (103/265 = 0.389) are involved in this pathway, with a *P* value of 1.25E-10 and a z-score of −2.524, suggesting relative inhibition in the early-onset group compared with late-onset one. The lower panel of Figure 6A is a volcano plot that demonstrates a positive fold expression of ROCK/MYLK-related genes in patients with early-onset POAG. In Figure 6B, although there is no variance in RHOA expression between early-onset and late-onset POAG, we observed significant upregulation of downstream kinases, such as ROCK1, ROCK2, and MYLK, as well as CALM2, in early-onset POAG (*P* < 0.05, *n* = 5). These proteins trigger the upregulated expression of p-MLC, enhancing actin-myosin interactions that are essential for cell contraction. Additionally, our results indicate that MYL6, which encodes a myosin light chain, is significantly elevated in early-onset POAG (*P* < 0.05, *n* = 5). The phosphorylation of MYL6 contributes crucially to the contractile machinery within the TM, positioning it as a potential driver of POAG pathogenesis. Late-onset POAG may not be as important a driver in the ROCK/MYLK pathway (see Fig. 7) as early-onset POAG. Although MYL6, both MLC and pMLC, is highly abundant in early-onset POAG, further differentiation must be made whether it is in a phosphorylated state or not. It is difficult to say whether this gene is associated with early or late onset POAG. It is important to consider the limitations of RNA expression and the need for further validation using techniques like immunohistochemistry (IHC) to confirm the findings.

## Discussion

Among the available glaucoma animal models, we successfully established microbead-induced OHT model of NZW rabbits using a single injection of microbeads, without causing damage to the anterior segment structure. Our methodology involved several modifications: (1) we used magnetic particles without any functionalized coatings, which minimized unwanted molecular interactions and eliminated the need to remove coating material prior to injection; (2) we utilized the superparamagnetic property of the beads to prevent aggregation or migration once attracted by a hand-held magnet; and (3) the utilization of a donut-shaped external magnet ensured the even distribution of the beads, thereby avoiding clumping that could compromise the effectiveness of IOP elevation.

Rabbits have increasingly become the preferred choice for many ophthalmic studies due to their practicality, cost-effectiveness, ethical considerations, and anatomic similarities to humans. The microbead-induced OHT rabbit model, which increases IOP by obstructing the outflow of AH, is a widely used experimental tool. This model effectively simulates key aspects of glaucoma, especially the mechanical obstruction of the AH outflow facility, making it highly relevant for studying the consequences of increased IOP. Although this microbead-induced OHT rabbit model does not fully mirror the pathology of human TM in POAG, our findings confirm that microbead-induced OHT rabbit can activate the ROCK/MYLK pathway (see Fig. 7) in the same way as early-onset POAG. When establishing glaucoma models in rabbits, it is essential to carefully consider the differences in AH dynamics, anterior chamber angle, and TM between humans and rabbits. Factors such as the narrow iridocorneal angle, the relatively underdeveloped TM, and the absence of Schlemm’s canal must be taken into account.^23^^,^^24^ Although the rabbit model may not fully replicate the histological and pathological features of the human TM and anterior chamber angle, our results indicate that activation of kinases, such as ROCK and MYLK, leads to the p-MLC, resulting in TM contraction. This mechanism aligns with the effects observed in the microbead-induced OHT rabbit model, suggesting that this model is partially suitable for studying certain dynamics of early-onset POAG, particularly where these specific kinases are implicated.

The microbead-induced OHT model of NZW rabbits we established not only manifested with a mean baseline IOP > 35 mm Hg but also demonstrated higher protein expression levels of pMLC-2 and MYLK-4, indicative of the activation of the ROCK/MYLK pathway. In our investigation into IOP-lowering strategies, we utilized commercially available ROCK inhibitors (AR-13324),^25^^,^^26^ alongside with a novel dual kinase inhibitor, ITRI-E-(S)4046, which targets both the ROCK and MYLK pathways. Although ROCK inhibitors have shown promising advances in treating glaucoma, their side effect profiles are markedly influenced by the potent inhibition of the multiple kinase pathway, which induces vasodilation leading to conjunctival hyperemia. For example, with 0.02% AR-13324 eye drops used once daily, 89% of patients (16 out of 18) experienced hyperemia, with most cases rated as mild.^27^ Similarly, ripasudil's 0.04% eye drops triggered a rapid onset of moderate hyperemia that quickly resolved.^28^ In contrast, the dual kinase inhibitor ITRI-E-(S)4046, which specifically targets both ROCK and MYLK4 (kinase profile)^17^^,^^18^ and minimally impacts tyrosine kinase (TK) activity, showed only transient and slight hyperemia at a concentration of 0.1% in NZW rabbits. This reduced side effect profile is likely due to the decreased activity of the TK enzyme, indicating a potentially different mechanism or side effect profile,^17^ and significantly reduced conjunctival dispersion—the 0.1% ITRI-E-(S)4046 formulation exhibits 7 times less conjunctival dispersion compared to 0.02% AR-13324 (data not shown).

Using our NZW rabbit OHT model, we assessed the effects of these inhibitors on modulating the activation of the ROCK/MYLK pathway. Our findings revealed that both 0.05% and 0.1% ITRI-E-(S)4046 exhibited superior reduction in IOP compared to 0.2% AR-13324 in the NZW rabbit OHT model. Notably, 0.1% ITRI-E-(S)4046 decreased the protein expression levels of pMLC-2 and MYLK-4, and expanded the percentage of space area, facilitating increased AH outflow facility and resulting in a reduction in IOP. The dual kinase inhibitor, (ITRI-E-(S)4046), can simultaneously effectively target both the ROCK and MYLK pathways, providing more effective modulation of the TM obstructed molecular pathways and deactivating ROCK/MYLK in our NZW rabbit OHT model.

In our clinical investigation, we used RNA sequencing to delve into the intricate workings of the ROCK/MYLK signaling pathway (see Fig. 7) in patients afflicted with early- and late-onset POAG. Our analysis, based on different onset POAG, unveiled differential gene expression patterns in the Rho family GTPase pathways, notably spotlighting the heightened (colored red) activity of genes including MYLK, and ROCK1/2, which are main characts in the ROCK/MYLK signaling pathway, as depicted in Supplementary Figure S8. The Rho family GTPase pathway elucidates a series of events triggered by extracellular matrix (ECM) deposition, and actin polymerization, and apoptosis.^29^^–^^31^ Among them, the activation of Rho triggers the activation of ROCK and MYLK, which phosphorylates MLC and inhibits MLCP,^32^ key steps in promoting myosin contraction. Thus, phosphorylated MLC-2 (pMLC-2) and MYLK-4 play integral roles in mediating the activation of ROCK/MYLK signaling on cellular responses to ECM cues, thereby influencing physiological processes like smooth muscle contraction within the TM. This signaling pathway's dysregulation has been implicated in the pathogenesis of various conditions, including glaucoma. Deactivating the ROCK/MYLK pathways, through the use of ROCK or ROCK/MYLK (dual kinase) inhibitors, can help reverse the resistance of AH outflow and reduce IOP by enlarging the void space of the TM.

It is worth mentioning that in the 5-to-5 PCA analysis depicted in Figure 6A, the early-onset POAG group exhibits a denser distribution of gene expression compared to the late-onset POAG group, indicating that early-onset POAG may be more influenced by genetic factors, whereas late-onset POAG may involve a combination of aging, genetic factors, and other environmental factors. Additionally, previous studies have shown that with aging, the proportion of uveoscleral outflow in the AH drainage decreases from 38% at ages 20 to 30 years to 3% at over 60 years old, with a significant increase in TM outflow burden by 35%.^33^ This indicates that the influence of the TM route on AH outflow cannot be overlooked in both early- and late-onset POAG cases. Our findings suggest potential therapeutic interventions for lowering IOP, particularly through the use of a ROCK/MYLK dual inhibitor in our TM-obstructed OHT model of NZW rabbit models and early-onset POAG.

## Conclusions

The establishment of reproducible IOP elevations in the OHT model of NZW rabbits provides a valuable platform for advancing our understanding of the biology and cellular mechanisms underlying glaucoma. This model enables targeted investigations into the TM-obstructed AH efflux pathway, offering insights crucial for the development of novel therapeutic strategies. In parallel, our clinical investigations utilizing next-generation sequencing have unveiled TM-related pathogenic pathways associated with patients with different onset POAG. Notably, pathways related to Rho signaling have emerged as critical components across various onset of POAG. Most importantly, the high abundance of ROCK1, ROCK2, MYLK, and CALM2 in patients with early-onset POAG suggests that these proteins are actively involved in the disease mechanism, particularly through their role in myosin light chain phosphorylation and subsequent cellular contractions that elevate IOP and may be a key contributor to the occurrence of early-onset POAG. The identification of these pathways underscores their significance in disease progression and highlights their potential as therapeutic targets. Among emerging two modalities, ROCK inhibitors represent a promising class of drugs for POAG management. However, currently available options are limited, and concerns regarding side effects persist. In response, the development of second-generation dual-kinase inhibitors, exemplified by ITRI-E-(S)4046, aims to address these limitations by offering enhanced IOP-lowering efficacy while minimizing adverse effects. This ongoing development holds considerable promise for improving the clinical management of POAG, providing patients with safer and more effective treatment options.

## Supplementary Material

Supplement 1
