# Supplementary material for: Activation of the ROCK/MYLK Pathway Affects Complex Molecular and Morphological Changes of the Trabecular Meshwork Associated With Ocular Hypertension
Source: Invest Ophthalmol Vis Sci. 2024 Aug 8;65(10):17. doi: 10.1167/iovs.65.10.17 (PMC11314630; doi:10.1167/iovs.65.10.17)
Supplement: Supplement 1 [file iovs-65-10-17_s001.pdf]

Activation of the ROCK/MYLK pathway affects complex molecular and morphological changes of the trabecular meshwork associated with ocular hypertension

Short Title: Activation of the ROCK/MYLK for ocular hypertension

Chia-Chen Hsu[1], Fang Pai Lin[2], Hao-Chen Tseng [2], Pin Kuan Ho [3], Yi-Hsun Chen[4], Yann-Guang Chen[1], Da-Wen Lu [1], Yi-Hao Chen[1], Jian-Liang Chou[5], Hsin-Chih Chen[5], Yu Chuan Huang[2,5*]

[1] Department of Ophthalmology, Tri-Service General Hospital, National Defense Medical Center, Taipei, Taiwan.

[2] School of Pharmacy, National Defense Medical Center, Taipei, Taiwan.

[3] School of Dentistry, National Defense Medical Center, Taipei, Taiwan.

[4] Biomedical Technology and Device Research Laboratories, Industrial Technology Research Institute, Hsinchu, Taiwan.

[5] Department of Research and Development, National Defense Medical Center, Taipei, Taiwan.

*Corresponding Author

Yu Chuan Huang, PhD

School of Pharmacy, National Defense Medical Center, Taipei, Taiwan

No.161, Sec. 6, Minquan E. Rd., Neihu Dist., Taipei City, 114201 Taiwan (R.O.C.). Tel: 886-9-66583837; Fax: 886-2-87923111

E-mail: yuh@mail.ndmctsgh.edu.tw

Supplementary Methods:

1. The preparation of experimental rabbits

(A) Rabbits' handling:

Two-month-old NZW rabbits, sourced from Taiwan and housed in the Laboratory Animal Center of National Defense Medical Center, were utilized in this study. The rabbits, weighing between 2.4 and 3.5 kg, were kept at room temperature (~24 °C) under a 12-hour light and 12-hour dark cycle, with ad libitum access to food. For the intracameral microbead-injection, the rabbits were sedated by intramuscular injection with 0.2 ml per kilogram of body weight of a reconstituted Zoletil50 solution containing 25 mg tetamine hydrochloride and 25 mg zolazepam hydrochloride (Zoletil® 50, Virbac, Suffolk, UK). For IOP measurements, rabbits were conscious and their cornea treated with topical anesthetic 0.5% proparacaine hydrochloride drops (Alcon®, ALCAINE, Puurs, Belgium). IOP was measured in each eye using a Model

30™ Pneumatonometer (Reichert, NY, USA), an applanation tonometer (Figure S1), with an average of 5 recordings per eye.

(B) The planar tonometer:

Our method use the Reichert® Model 30™ which is recognized for its accuracy compared to non-contact tonometry, provided reliable measurements by flattening a smaller corneal area (5 mm^2) compared to the Goldmann tonometer (7.5 mm^2). This smaller contact area reduces the effects of corneal stiffness, tear surface tension, and corneal thickness on measurements. In addition, we performed calibration using a calibration verifier (15 ± 2 mmHg) according to the manufacturer's instructions. The manufacturer does not provide separate calibration protocols for different species, which supports the use of standardized methods in studies. In 2016, Salvi et al., applied a Reichert® Model 30™ in rabbits [Valvi et al., 2016; PMID: 27092593] to measure IOP in New Zealand normotensive male albino rabbits through a planation tonometry method. Our previous study, published in IOVS [Chen et al., 2021; PMID: 34661609], demonstrated the repeatability and reproducibility of the Reichert® Model 30™ Pneumatonometer in a NZW rabbit model, confirming its reliability.

Reference:

1. Salvi A, Bankhele P, Jamil J, Chitnis MK, Njie-Mbye YF, Ohia SE, Opere CA. Effect of Hydrogen Sulfide Donors on Intraocular Pressure in Rabbits. J Ocul Pharmacol Ther. 2016 Jul-Aug;32(6):371-5. doi: 10.1089/jop.2015.0144. Epub 2016 Apr 19. PMID: 27092593.
2. Chen YH, Lin WY, Huang YC, Ho WY, Fu CW, Tu CM, Hwang CS, Hung CL, Lin MC, Cheng F, Wang YJ, Chen CH, Chou SH, She MP, Yang CY, Cheng HL, Liu CP, Lu DW. The Intraocular Pressure Lowering Effect of a Dual Kinase Inhibitor (ITRI-E-(S)4046) in Ocular Hypertensive Animal Models. Invest Ophthalmol Vis Sci. 2021 Oct 4;62(13):12. doi: 10.1167/iovs.62.13.12. PMID: 34661609.

2. The procedure for inducing OHT in rabbits:

After manually vortexing the bottle and suspending the microbeads evenly, magnetic microbeads were extracted using a 1-mL syringe with a 26-gauge needle and then replacing the 26-gauge needle with a 27-gauge needle for injection. A 27-gauge needle was selected for injection based on pilot studies, which revealed that microparticles impeded by a 30-gauge needles and that a needle larger than 27-gauge would lead to corneal wound leakage. A Jameson muscle hook (Weck®, Morrisville, NC, USA) was used to fix and push out the OD of the rabbits. Simultaneously, an intracameral injection through the limbus with the needle bevel

up was performed. Before injecting microbeads, it is recommended to extract a space-safe amount of AH to prevent increase in IOP from the added volume. We pulled the needle outwards by 1–2 mm to release the AH, then inserted it back carefully to administer 0.1 mL of magnetic microbeads over a duration of 15–20 seconds to avoid mechanical injury to the anterior segment of the eye. The customized neodymium magnet was placed in the center of the eye to fit around the rabbit's eyeball. During the injection, the customized donut-shaped neodymium magnet was used to attract and distribute the magnetic microbeads evenly into the iridal angle. The magnet was kept there for 15–20 min after the retraction of the needle to prevent the backflow of microbeads. The puncture wound was left to recover naturally, and an anti-infective ophthalmic solution, 0.5% moxifloxacin (NORVATIS, Fort Worth, TX, USA), was topically applied to prevent infection.

3. Immunoscore for MYLK4 or p-MLC2 identified by INHAND:

On day 14, post-magnetic microbead-injected, TM tissue was dissected from the anterior segment and fixed. Rabbit polyclonal antibodies specific for pMLC-2 (Thr18/Ser19) (1:200, Cell Signaling TECHNOLOGY, Taipei, Taiwan) and MYLK-4 (1:200, Antibody [NBP1-80761]; Novus Biologicals, Centennial, CO, USA,) were used to tag pMLC-2 and MYLK-4 in brown, respectively. The

expression level of pMLC-2 and MYLK-4 was assessed according to the International Harmonization of Nomenclature and Diagnostic Criteria (INHAND). The expression intensity was scored from 0–5 as follows: (0) none, (1) minimal, (2) mild, (3) moderate, (4) moderately-high, and (5) severe.

4. Hematoxylin and eosin (H&E) examination:

14 days after intracameral microbead injection, magnetic microbead distribution in the anterior chambers was assessed. The animals were euthanized with carbon dioxide inhalation whilst being under anesthesia. Both the induced and control eyes were removed and the anterior segment tissue was fixed, processed, embedded, and cryosectioned. Hematoxylin and eosin (H&E) was then used to histologically stain the slides for microscopic examination. Microscopic imaging (Leica DM6B; Leica Microsystems Ltd., Milton Keynes, UK) was performed using a digital camera (Leica DFC450C, Leica Microsystems Ltd, Wetzlar, Germany).

5. The bioinformatic analysis of RNAseq data:

After the raw reads obtained by sequencing were filtered, CLC Genomics Workbench 23.0.5 was used to map the clean reads to the human genome (GRCh38) and DEGs' analysis. Use Principal Component Analysis (PCA) (PC1:47% and PC2:17% variance) to look for clusters or patterns in the 2D representation to

differentiate patients with early-onset POAG from those with late-onset POAG. Clustering may indicate that individuals with similar genetic characteristics are grouped together. By visualizing the first two principal components (PC1 and PC2) of an individual, we can gain insight into the underlying genetic structure and subpopulation differentiation within the POAG population. This may suggest potential genetic factors associated with early or late onset of POAG. Subsequently, we formatted the differential expression genes into an Excel spreadsheet and imported it into Ingenuity Pathway Analysis (IPA) for core analysis. Canonical Pathways likely included identifying significantly affected pathways, networks, and biological processes associated with the observed differential gene expression in the case group compared to the control group, with the results presented in a bar chart. The findings from IPA were interpreted in the context of the known functions and interactions of the Rho family of GTPases in cellular processes and disease pathways. This analysis helped to elucidate potential genetic factors associated with early or late onset of POAG.

Supplementary Figures:

Figure S1. Intraocular pressure measurement in rabbits.

Intraocular pressure of awake New Zealand White (NZW) rabbits were measured using a Model 30™ Pneumatonometer (Reichert, NY, USA) with a neck restraint by hand.

Figure S2.

The histiocytes were identified by their distinctive morphological characteristics in the IHC staining of MYLK4.

Panel (A): These cells are primarily characterized by their round shape and small, kidney-shaped nuclei, which strongly indicate that they are histiocytes (indicated by arrows). Panel (B): We applied a consistent threshold across all experimental figures to systematically assess these cells and segment the cells with the strongest staining. The results of this segmentation are presented, where we quantified the histiocytes for statistical evaluation. Panel (C): Areas that did not contain tissue were labeled 'trabecular meshwork space'. Panel (D): The remaining stromal tissue was analyzed to evaluate the immunoscore for MYLK4 staining.

Figure S3. Distribution of magnetic microbeads in the anterior segment following injection.

(A) The microbeads attracted by a rod-shaped magnet clustered in 160 degrees of iridocorneal angle.

(B) The magnetic microbeads were attracted iridocorneal angle with an even 360-degree distribution by the toroidal magnet held around eye.

Figure S4. Quantification of pMLC-2 intensity in control and ocular hypertensive eye. Immunohistochemical stainings of phosphorylated myosin light chain (pMLC-2) of the TM from non-injected eyes and microbead-injected OHT eyes using pMLC-2 (Thr18/Ser19) antibody (Cell Signaling Technology, Danvers, MA, USA)). Expression intensity of pMLC-2 was scored according to International Harmonization of Nomenclature and Diagnostic Standard (INHAND) (Supplementary methods 3) and was presented as mean scores. Result revealed that the total scores was significant higher in microbead-injected than the corresponding control eyes. Data are mean score of four microbead-injected eyes and three uninjected eyes. *$p<0.05$.

Figure S5 Quantification of MYLK-4 intensity in control and ocular hypertensive eye. Immunohistochemical stainings of myosin light chain kinase 4 (MYLK-4) of the TM from non-injected eyes and microbead-injected OHT eyes using MYLK4-polyclonal antibody (NOVUS, #NBPI-80761). Expression intensity of MYLK4 was scored according to International Harmonization of Nomenclature and Diagnostic Standard (INHAND) (Supplementary methods 3) and was presented as mean scores. Result

revealed that the total scores was significant higher in right microbead-injected eye than the fellow uninjected eye. Data are mean score of three microbead injected eyes and four uninjected eyes. *$p<0.05$.

Figure S6 Hematoxylin and eosin stained sections of iridocorneal angle.

(A) Histological section of magnetic-microbead-injected eye shows infiltration of magnetic bead in the trabecular meshwork.

(B) Histological section of trabecular meshwork from control eye. (H&E x20). MB, microbeads; TM, trabecular meshwork.

Figure S7 Ingenuity Pathway Analysis (IPA) pathways: it presents a bar chart illustrating pathway analysis results, with the x-axis showing the negative logarithm of the p-value, emphasizing significance. The chart is arranged to highlight the most significant pathways. Orange bars denote predicted pathway activation, blue bars indicate predicted inhibition, and gray bars represent pathways with uncertain activity prediction. White bars signify pathways with z-scores near 0 or insufficient molecules for analysis. The Rho family GTPase pathway in this bar chart shows that 103 DEGs out of 265 molecules (103/265=0.389) are involved in this pathway, with a p-value of 1.25E-10 and a z-score of -2.524, suggesting relative inhibition in the early-onset group

compare with late-onset one.

Figure S8 Rho family GTPase pathways from IPA (Ingenuity Pathway Analysis). We imported the 5-to-5 differential expression data into IPA to generate the diagram from Rho family GTPase pathway. In the pathway, red/green denotes increased/decreased measurements, and orange/blue indicates predicted activation/inhibition. The early-onset POAG patients showed a heightened expression trend associated with actin contraction, potentially due to increased ROCK1/2, MYLK, CALM2 expression promoting pMLC activation. It suggests a pathologically significant increase in TM contractility, possibly influencing AH outflow facility. Furthermore, our investigation into the molecular cross-talk within the Rho family GTPase pathway, especially between the ROCK/MYLK pathways and other pivotal signaling molecules such as RAC1, CDC42, cadherins (CDH), and integrins, is set to significantly advance our understanding of TM cellular dynamics. This research is critical for elucidating the intricate regulatory networks that govern cell survival, proliferation, and differentiation within the TM.

Figure S1

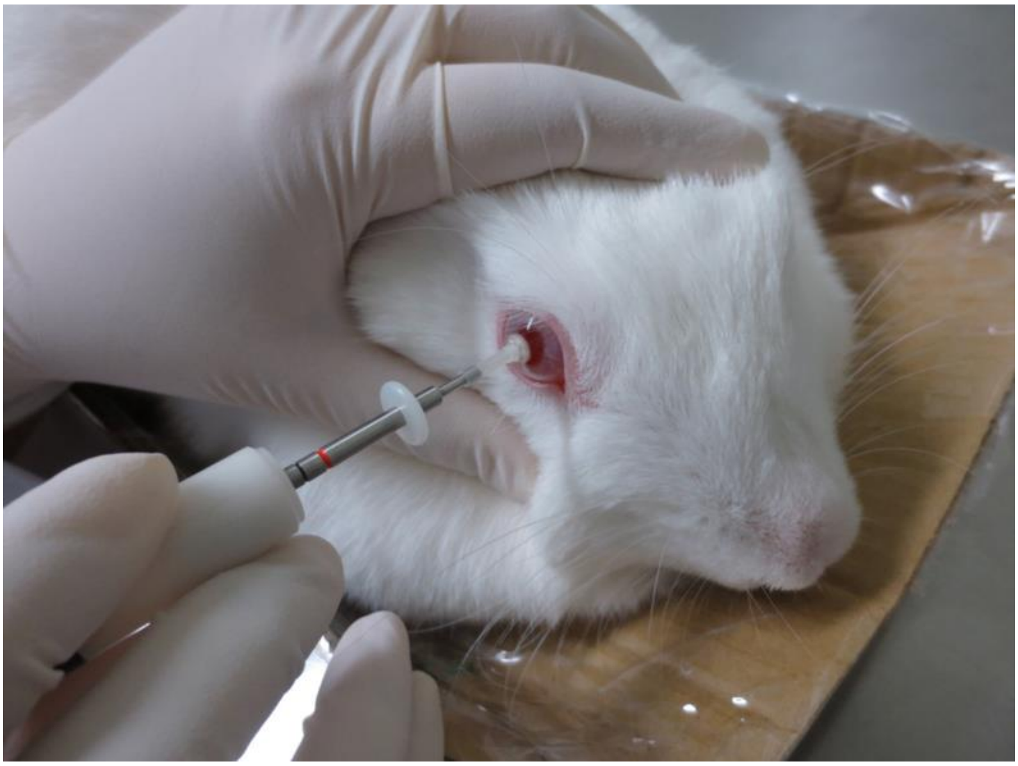

Figure S2

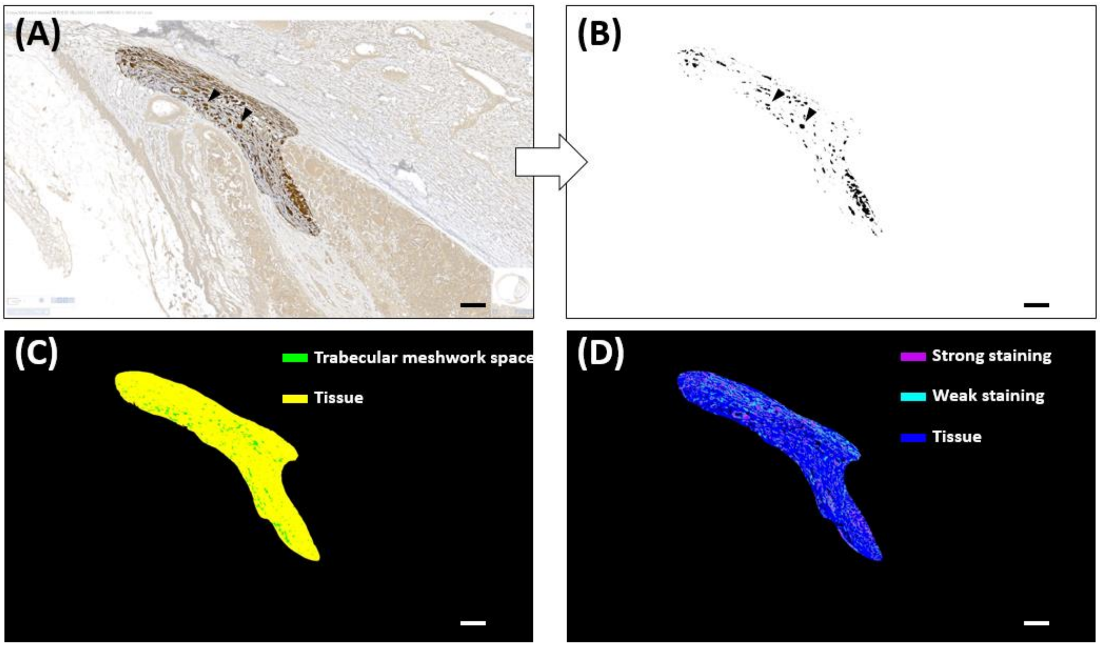

Figure S3

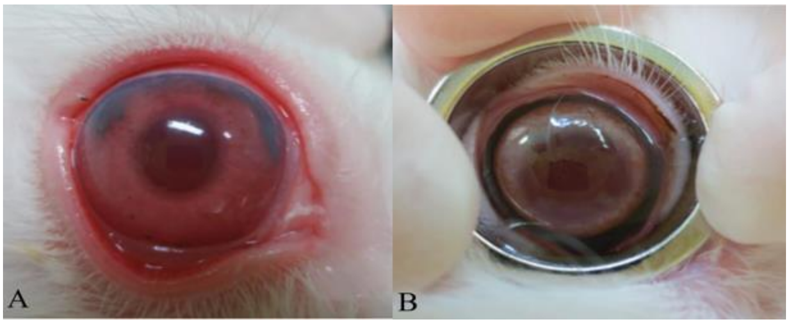

Figure S4

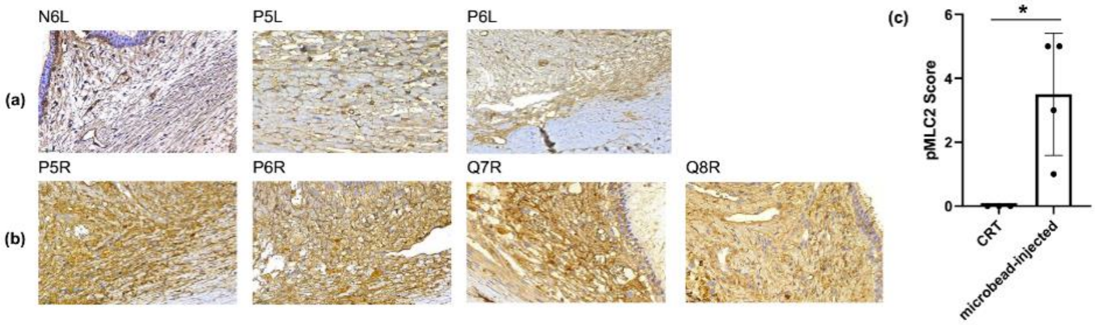

Figure S5

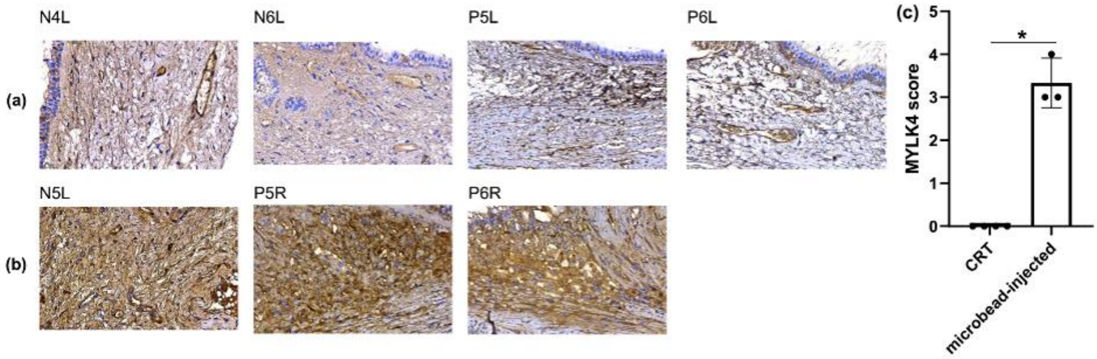

Figure S6

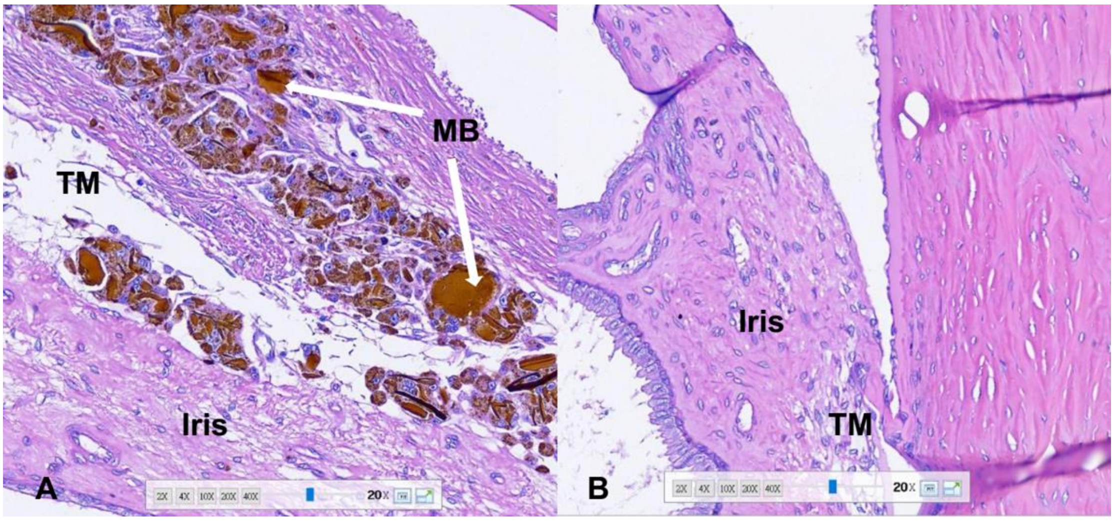

Figure S7

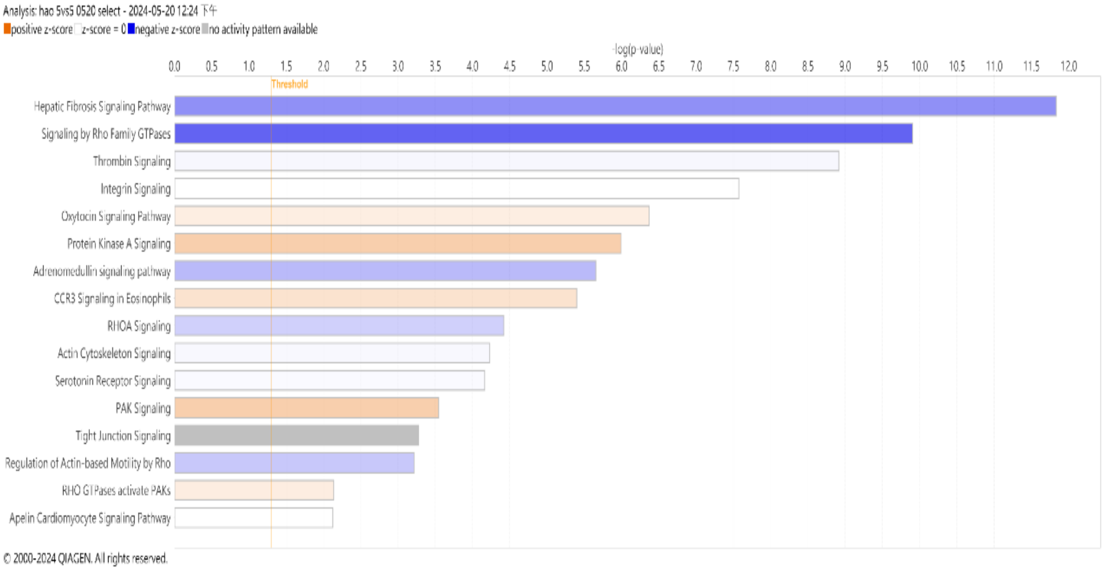

Figure S8

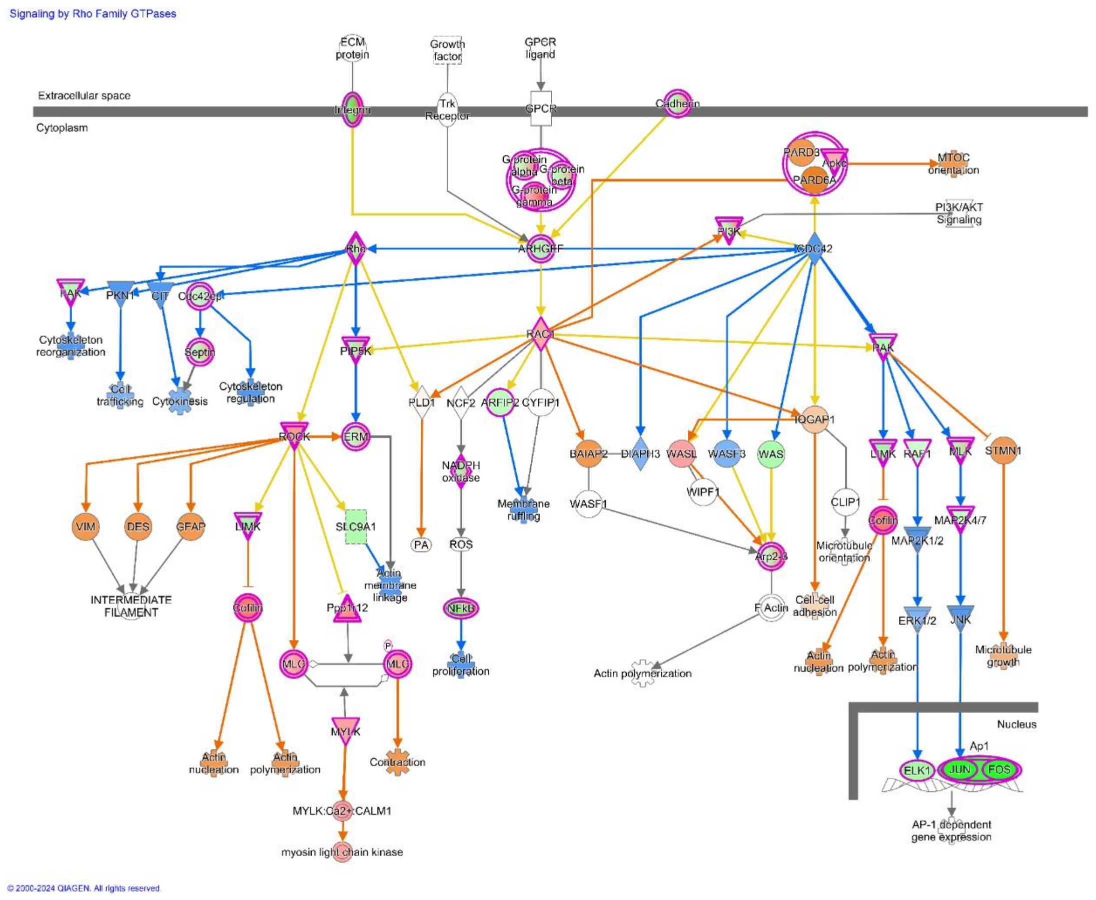

Signaling by Rho Family GTPases
ECM protein
Growth factor
GPCR ligand
Extracellular space
Cytoplasm
Integrin
Trk Receptor
GPCR
Cadherin
G-protein alpha
G-protein beta
G-protein gamma
PARD3
Apkc
PARD6A
MTOC orientation
PI3K/AKT Signaling
PI3K
CDC42
Rho
ARHGEF
PAK
PKN1
CIT
Cdc42ep
Cytoskeleton reorganization
Septin
Cell trafficking
Cytokinesis
Cytoskeleton regulation
RAC1
PIP5K
PLD1
NCF2
ARFIP2
CYFIP1
IQGAP1
ROCK
ERM
NADPH oxidase
Membrane ruffling
BAIAP2
DIAPH3
WASL
WASF3
WAS
LIMK
RAF1
MLK
STMN1
WASF1
WIPF1
CLIP1
VIM
DES
GFAP
SLC9A1
Cofilin
MAP2K4/7
MAP2K1/2
Microtubule orientation
PA
ROS
Arp2/3
INTERMEDIATE FILAMENT
Actin membrane linkage
Ppp1r12
NFkB
F Actin
Cell-cell adhesion
ERK1/2
JNK
MLC
P
Cell proliferation
Actin polymerization
Actin nucleation
Microtubule growth
Nucleus
MYLK
Contraction
Ap1
ELK1
JUN
FOS
AP-1 dependent gene expression
MYLK:Ca2+:CALM1
myosin light chain kinase
© 2000-2024 QIAGEN. All rights reserved
